# Phenolic Compounds and Antioxidant Activities of Potato Cultivars with White, Yellow, Red and Purple Flesh

**DOI:** 10.3390/antiox8100419

**Published:** 2019-09-20

**Authors:** Weidong Ru, Yuehan Pang, Yuanruo Gan, Qin Liu, Jinsong Bao

**Affiliations:** 1 Institute of Nuclear Agricultural Sciences, Key Laboratory for Nuclear Agricultural Sciences of Zhejiang Province and Ministry of Agriculture and Rural Affairs of China, Zhejiang University, Zijingang Campus, Hangzhou 310058, China; 21716010@zju.edu.cn (W.R.); pyh_tt@163.com (Y.P.); 2 College of Food Science and Engineering/Collaborative Innovation Center for Modern Grain Circulation and Safety/Key Laboratory of Grains and Oils Quality Control and Processing, Nanjing University of Finance and Economics, Nanjing 210023, China; tomogyr@163.com (Y.G.); qinliu@nufe.edu.cn (Q.L.)

**Keywords:** antioxidant activity, bound fraction, color, phenolics, phenolic acids, potato

## Abstract

The contents of total phenolics (TPC), individual phenolic acid and antioxidant activities in the free and bound fractions of potato with different flesh colors were systematically investigated. The TPC and antioxidant capacity in the bound fraction was significantly lower than that in the free fraction. Chlorogenic acid, neochlorogenic acid, cryptochlorogenic acid, caffeic acid, *p*-coumaric acid and ferulic acid were detected in the free fraction with chlorogenic acid being the most predominant, accounting for 35.21–81.78% of the total content. Caffeic acid, *p*-coumaric acid and ferulic acid were detected in the bound fraction in the colored potato with caffeic acid being the major one. In the free fraction, the content of each individual phenolic acid had positive correlation with antioxidant activity. In the bound fraction, caffeic acid and *p*-coumaric acid showed positive correlation with antioxidant activity. This study promotes further understanding of the correlations among TPC, phenolic acids and antioxidant activity.

## 1. Introduction

Potato (*Solanum tuberosum* L.) is the sole major tuber crop and the fourth most important food crop worldwide [1,2,3]. The total world potato production was estimated at 388,191,000 t in 2017 and almost a third of all potatoes were harvested in China and India [4]. The nutritional quality of potato is important to human health whose staple food is potato [5]. The potato contains minerals, proteins and antioxidant compounds such as carotenoids, phenolics, flavonoids, vitamins C and E, among others [5,6,7]. These phytochemicals in potato have attracted more and more attention from the public because of their effects on promotion of physical well-being [2,8].

Wide diversity in the phytochemicals and nutritional compositions in potato has been reported [9]. For example, the free phenolic components in potato have been widely investigated. Ramamurthy et al. reported that the chlorogenic, neochlorogenic, cryptochlorogenic, *p*-coumaric and ferulic acids were detected in potato [10]. Im et al. reported that the caffeic acid, chlorogenic acid, cinnamic acid, ferulic acid, gallic acid, and protocatechuic acid were richly present in potato peel [11]. Xu et al. [12], Albishi et al. [13] and Kim et al. [14] reported that chlorogenic, neochlorogenic, cryptochlorogenic, caffeic, *p*-coumaric, ferulic, vanillic acids, quercetin glucoside and tryptophan were detected in potato flesh. Although the contents of the different phenolic components were varied in different reports, chlorogenic acid was the most abundant [11,12,13,14]. The variation in phenolic acids may be due to different genotypes, and different extraction and analytical methods.

Phenolic acids and flavonoids are the most common phenolic compounds, which are generally present in both free and bound forms [15]. Chu et al. reported the free and bound phenolic content of 10 kinds of vegetables, and found that free phenolic acids accounted for 60.1%, 62.4%, 67.1% and 73.8% of the total phenolic acids in potato, carrot, cabbage and cucumber [16]. However, most of the studies only investigated the free phenolics based on the soluble extraction and neglected the bound phenolics, so the total phenolic content (TPC) of potato and antioxidant activities were underestimated in those works, especially for the colored potato. Anthocyanins are one kind of flavonoid, which are present in the colored potato and exhibit a range of biological activities [17,18,19]. Nemś et al. indicated that pelargonidin derivatives were present in greatest quantity in the red-fleshed potatoes, and petunidin and malvidin were the most abundant anthocyanins in the purple potatoes [20].

Although many assays have been conducted to determine the phenolic acids, anthocyanins and antioxidant capacity in potato, the individual phenolic acid composition and antioxidant capacity in the bound form were rarely reported. Furthermore, the relationships between the content of individual phenolic acid, TPC and antioxidant capacity have not been clearly understood.

The objectives of this study were: (1) to investigate the composition and levels of phenolic acids, and the antioxidant capacity of both free and bound fractions of potato accessions; (2) to analyze the relationships between the content of individual phenolic acid and TPC and antioxidant activities; and (3) to assess the composition of anthocyanin and total anthocyanin contents from red and purple flesh potatoes. The results from this study may further contribute to our understanding of the genetic diversity in the phenolics and processing techniques to benefit the desired nutritional qualities of potato crops.

## 2. Materials and Methods

### 2.1. Sample Preparation

The 14 potato cultivars were planted and harvested in the Zhejiang University farm in Hangzhou, China, in 2016 (Table 1) [1]. After harvesting, representative samples were obtained by selecting around 15 tubers with similar size for each cultivar, which were washed and peeled manually. Then they were cut to pieces and quickly stored at −80 °C. The potato pieces were put in a freeze dryer for 2 days at −50 °C. The dried potato samples were ground into powder and stored at 4 °C until analyzed.

### 2.2. Chemicals

Phenolic acid standards (caffeic acid, chlorogenic acid, *p*-coumaric acid, cryptochlorogenic acid, ferulic acid and neochlorogenic acid), Folin–Ciocalteu reagent, sodium hydroxide, 6-hydroxy-2,5,7,8-tetramethylchroman-2-carboxylic acid (Trolox), 1,1-diphenyl-2-picrylhydrazyl radical 2,2-diphenyl-1-(2,4,6-trinitrophenyl) hydrazyl (DPPH), 2,2-azino-bis-3-ethylbenzothiazoline-6-sulphonic acid diammonium salt (ABTS), and gallic acid were purchased from Sigma-Aldrich Chemical Co. (St. Louis, MO, USA). Methanol (HPLC grade) was bought from the Fisher Scientific Co. (Ottawa, ON, Canada). Acetic acid (HPLC grade) was bought from Macklin (Shanghai, China). Ethyl acetate, hexanes, hydrochloric acid, potassium chloride, potassium persulfate, sodium acetate, sodium carbonate, and sodium sulfate were purchased from the Sinopharm Chemical Reagent Co., Ltd. (Shanghai, China).

### 2.3. Extraction of Free/Soluble Phenolics and Bound Phenolics

The extraction method of free/soluble phenolics was performed according to the procedure described by Pang et al. [21]. Briefly, one gram potato powder (dry basis) was first defatted with 10 mL of hexanes. Then potato powder was extracted two times using 20 mL of 80% methanol at room temperature. Each time, the extraction mixture was shaken for 30 min. Then, the mixture was subsequently centrifuged at 8000× *g* for five min. After accomplishing centrifugation, the supernatants were collected and the pH adjusted to 1.5–2.0. The supernatant was then concentrated by a rotary evaporator (IKA RV10 digital V, Staufen, Germany) at 37 °C. The concentrated fraction was extracted with 60 mL ethyl acetate three times. The pooled extracts were evaporated with the same rotary evaporator at 35 °C, then 5 mL of 50% methanol was used to dissolve the dried extracts, which were stored at –20 °C and used as crude free fraction for phenolic compounds analysis.

The solid residue after extracting soluble phenolics was used to extract bound phenolics. The residue was first digested with 20 mL of NaOH (4 M) for 2 h at room temperature. The pH was adjusted to 1.5–2.0 using concentrated HCl, the mixture was then extracted three times using 60 mL of ethyl acetate. The pooled ethyl acetate extractions were evaporated and dissolved in methanol according to the same procedures stated above for free extraction [21]. 

### 2.4. Total Phenolic Content (TPC)

The TPC was measured using the colorimetric method with Folin–Ciocalteu reagent [21,22]. The TPC was expressed as milligrams of gallic acid equivalent (mg GAE) per 100 g of potato powder.

### 2.5. Determination of DPPH• Radical and ABT•^+^ Radical Cation Scavenging Activities

The DPPH assay was accomplished with the procedures described by Yamaguchi et al. with minor modification [21,23]. Briefly, 200 μL of the diluted free or bound extracts were added to 3 mL DPPH• radical solution (100 μM), which was prepared in methanol. The reaction was kept in the dark at room temperature for 30 min, and a spectrophotometer was used to measure the absorbance at 517 nm. The DPPH scavenging activity was expressed with inhibition (percent) of DPPH• absorbance [21]. 

The assay of ABTS•^+^ radical cation scavenging activity was conducted according to the procedure of Re et al. with slight modification [21,22,24]. First, 7 mM ABTS and 2.45 mM potassium per sulfate was mixed at room temperature in dark for 20 h to generate ABTS•^+^ radical cation. Then, methanol was used to dilute the ABTS•^+^ mixture to an absorbance around 0.700 at 734 nm. Last, 3.9 mL of ABTS+ solution was added to 0.1 mL of the extracts which were first diluted appropriately. The reaction mixture was kept at room temperature for 6 min, then the absorbance was recorded at 734 nm by a spectrophotometer. 

Trolox (0.5 mM) was served as a reference antioxidant. The results of DPPH radical scavenging activities and ABTS•^+^ radical cation scavenging activities were expressed as µM of Trolox equivalents (TE) per 100 g of potato powder using a standard curve of Trolox.

### 2.6. Total Anthocyanin Content (TAC)

TAC assay was carried out using a pH differential method [25,26,27]. The anthocyanin was extracted with 0.5 g potato powder mixed with 15 mL methanol to 1 M HCl (85:15, *v/v*) three times under dark condition, and each time held on a shaker for 20 min. Then supernatants were collected using a centrifuge at 6000× *g* for 15 min at room temperature. The TAC was expressed as cyanidin-3-glucoside equivalent.

### 2.7. HPLC Analysis of Phenolic Acids

An HPLC system (Waters Associate, Milford, MA, USA) was used for separation of phenolic acids [21]. The 5 µL extraction was injected by an autosampler. The mobile phase consisted of 0.1% acetic acid in water (A) and 0.1% acetic acid in methanol (B), and the flow rate was 1 mL/min. A 45 min linear gradient was set as follows: 0–1 min, 10–15% B, 1–30 min, 15–18% B, 30–40 min, 18–40% B, 40–45 min, 40–10% B. The phenolic acids were detected at a wavelength of 280 nm. The content of individual phenolic acid was quantified using external calibration curve.

### 2.8. LC-MS/MS Analysis of Anthocyanin 

The phenolics were analyzed by a UPLC-TOF-MS/MS system, which consisted of an ultrahigh pressure chromatograph (UPLC) (Shimadzu Co., Ltd., Kyoto, Japan) and an ESI 5600 triple quadrupole time of flight (TOF) mass spectrometer (Sciex Co., Ltd., Framingham, MA, USA). A Zorbax SB-C18 column (3.5 μm, 2.1 × 100 mm) (Agilent Technologies, Inc., Palo Alto, CA, USA) was used for separation of the anthocyanins. The same injection volume and mobile phase as in separation of phenolic acids were used, but the flow rate was 0.5 mL/min. A linear gradient with the following proportions of solvent B was used: 0–1 min, 10–15% B; 1–10 min, 15–20% B; 10–15 min, 20–95% B; 15–18 min, 95–10% B. MS parameters were set as follows: capillary voltage, 2000 V; sample cone voltage, 30 V; source temperature, 150 °C; desolvation temperature, 250 °C; desolvation gas (nitrogen gas) flow rate, 900 L/h; data acquisition range, *m/z* 100–1000 Da; ionization mode, positive. The MS/MS spectra were acquired by using collision energy of 35 V. 

### 2.9. Statistical Analysis

The results were presented as mean ± standard deviation (SD), in which all the measurements were accomplished at least in duplicate. Duncan’s multiple range test of ANOVA and correlation analysis were conducted using the SPSS 20.0 software (SPSS, Inc., Chicago IL, USA).

## 3. Results and Discussion

### 3.1. TPC

The TPCs of free and bound extractions of potato flesh samples are presented in Table 1. The TPC of the free fraction in potato flesh was much higher than that in the bound fraction for every accession. The TPC of the free fraction in red or purple potatoes was 51.36–73.60 mg GAE/100 g while it was 8.77–19.91mg GAE/100 g in yellow or white potatoes. The TPC of the bound fraction in red or purple potatoes was 7.72–40.45 mg GAE/g while it was 2.63–6.14 mg GAE/100 g in yellow or white potatoes. These results indicated that TPC of the colored potatoes was much higher than that of ordinary potatoes. Albishi et al. reported that the TPC of free fraction in four potato varieties, including yellow and purple potatoes, ranged from 45 to 118 mg GAE/100 g, with the bound phenolic content ranging from 33 to 50 mg GAE/100 g [13]. These values were similar to our study, but in our study the bound TPC in colored potatoes was significantly higher than yellow or white potato (Table 1). 

Chu et al. reported that potato had the highest bound phenolics (39.9%) among 10 different vegetables [16]. In most studies, only free phenolics were extracted using aqueous methanol, ethanol or mixed extraction method [28], and the TPC of free fraction was determined without taking the bound fraction into consideration [7,14,29,30]. Our results showed that the bound phenolic content accounted for 10.5–39.4% of the total phenolic content, and the colored potatoes had higher bound phenolic content. In this study, 14 potato accessions were planted and harvested at the same time in the same field, and the growing conditions, cultivation techniques and extraction process were same, so the variation in TPC may be attributed to the diverse potato genotypes.

### 3.2. Antioxidant Capacity and Its Relation to the TPC

Albishi et al. argued that due to different mechanisms involved in the test of antioxidant capacity, the best conclusion could be reached when each research used at least two methods [13]. In view of this fact, the antioxidant activity of the extract was determined by two different methods (DPPH• and ABTS•^+^) in our study. The values of DPPH• and ABTS•^+^ radical scavenging capacity are displayed in Table 1. The DPPH• radical scavenging activities of free and bound fractions ranged from 21.76 to 278.33 and from 7.03 to 132.75 µM TE/100 g, respectively. The ABTS•^+^ radical cation scavenging activity of free and bound fractions ranged from 21.23 to 309.48 and from 0.64 to 124.49 µM TE/100 g, respectively. Among all the samples, the DPPH• and ABTS•^+^ radical scavenging activities of the bound fraction were much lower than those of free fraction. Reddivari et al. reported that higher antioxidant activity was found in the flesh and skin of purple potato genotypes [30,31]. Albishi et al. also reported purple potato flesh had higher DPPH• radical scavenging activities than yellow potato flesh, but the ABTS•^+^ radical scavenging activities of purple potato flesh were similar with those of the yellow potato flesh [13]. In our study, for either free fraction or bound fraction, the colored potato (PT11, PT13, PT14, PT18) had higher average levels of antioxidant capacity than the yellow and white potato flesh (Table 1). PT11 and PT18 had much higher antioxidant capacity, and even the antioxidant activity of the bound fractions of PT11 and PT18 were higher than that of free fractions of all yellow or white flesh potatoes.

In order to reveal the contribution of TPC to the antioxidant activity measured using DPPH and ABTS radical scavenging methods, the Pearson correlations between the TPC of free and bound fraction and total antioxidant activities were analyzed. The correlation coefficients of TPC, DPPH and ABTS in free and bound phenolics are shown in Table 2. 

All the pair-wise correlations were highly positive (Table 2). No matter what fractions, free or bound, the correlation coefficients between TPC, DPPH and ABTS were higher than 0.980. The correlations between free and bound fractions were also highly positive (*r* > 0.705, *p* < 0.01). The results indicated that the higher phenolic content would be accompanied by a higher antioxidant activity, and the higher free TPC would result in higher bound TPC. Compared with other literature, Reddivari et al. reported that a significant positive correlation was found between free TPC and antioxidant activity against both the DPPH radical (*r* = 0.93; *p* < 0.001) and the ABTS radical (*r* = 0.90; *p* < 0.001) [30]. Hesam et al. [32] and Albishi et al. [13] reported that there was no correlation between free TPC and DPPH, but found a highly positive correlation between bound TPC and total antioxidant activity (*r* = 0.97, *p* < 0.01) [13]. 

### 3.3. Identification and Quantification of Individual Phenolic Acid

**Figure 1:**
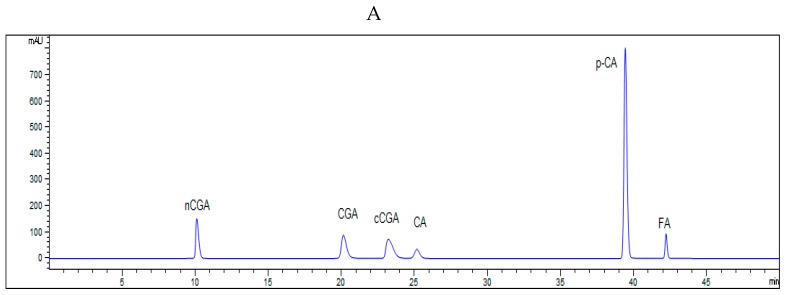

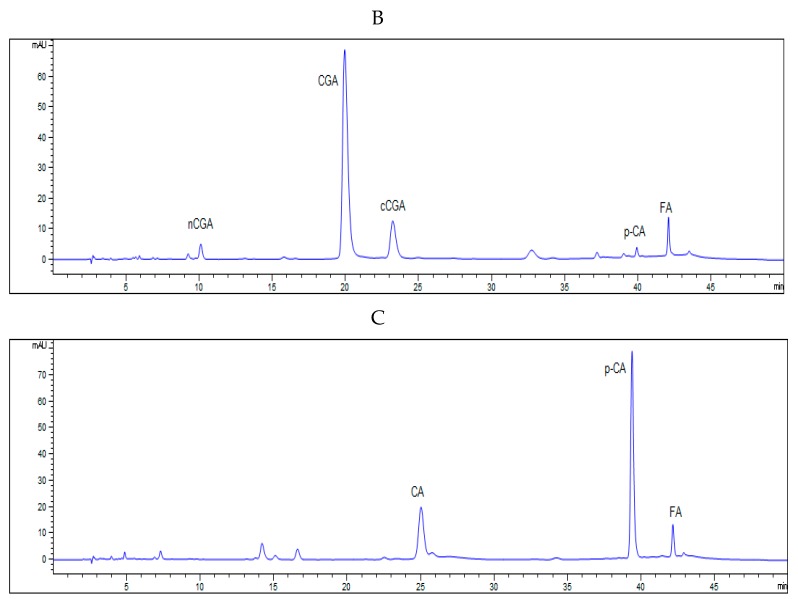
HPLC chromatogram of free and bound phenolic content of potato powder at 280 nm. (**A**) Phenolic acids standards. (**B**) The free phenolic acids of PT14. (**C**) The bound phenolic acids of PT11. CGA, chlorogenic acid; nCGA, neochlorogenic acid; cCGA, cryptochlorogenic acid; CA, caffeic acid; *p*-CA, *p*-coumaric acid; FA, ferulic acid.

The chromatograms of phenolic acids in the free and bound fraction of potato samples at 280 nm are shown in Figure 1. The predominant phenolic acids in the free fractions of all potato samples and in the bound fractions of the four colored potato genotypes are displayed in Table 3; Table 4, respectively. 

The caffeic acid, chlorogenic acid, *p*-coumaric acid, cryptochlorogenic acid, ferulic acid, and neochlorogenic acid were detected in most yellow and white flesh potatoes in free phenolic extraction. However, caffeic acid was not detected in PT29, and *p*-coumaric acid was not detected in PT29, PT34, PT36 and PT38. Chlorogenic, neochlorogenic, cryptochlorogenic, *p*-coumaric and ferulic acids were detected in the colored potatoes. It is noteworthy that caffeic acid was not observed in free fraction of four colored potatoes, which is in agreement with the result of Albishi et al. who also reported caffeic acid was not detected in free fraction of purple potato flesh [13]. Wide diversity in the phenolic acids profile in different potato genotypes has been reported in the literature [10,12,14]. Rojas-Padilia et al. even reported that chlorogenic acid, neochlorogenic acid, vanillin, *p*-coumaric acid, caffeic acid, coumarin, 4-hydroxy-3-methoxycinnamaldehyde and ferulic acid were detected in potato cooking water [33]. The diverse phenolic acids in different genotypes may be attributed to a complex biosynthesis pathway of phenolic acids in potato [34].

In the free fraction, the content of chlorogenic acid ranged from 2.06 to 79.91 mg/100 g DW, which was the highest in the most samples except for PT29 in which the ferulic acid content was the highest (Table 3). Chlorogenic acid accounted for 35.21% (PT05) to 81.78% (PT11) of free phenolic acids, while Reddivari et al. reported that it accounted for 50% to 70% of free phenolic acids [30]. The neochlorogenic acid and cryptochlorogenic acid are two isomers of chlorogenic acid. For free phenolics, cryptochlorogenic acid had the second highest content in PT11, PT13, PT14 and PT34; caffeic acid had the second highest content in PT10, PT28, PT35, PT36 and PT38; and ferulic acid had the second highest content in PT05, PT12, PT18 and PT30. The content variation of different phenolic acids supported the viewpoint that the differences in nutritional profile found in potato nutritional composition are due to its wide biodiversity [9].

In the bound fraction, no phenolic acid could be detected in the white and yellow potatoes, but caffeic acid, *p*-coumaric acid and ferulic acid were detected in the four colored potatoes (Table 4). Although chlorogenic acid was the highest content in free phenolics, it was not observed in bound phenolic fraction. Instead, the caffeic acid was the major phenolic acid in the bound fraction in colored potato samples, followed by ferulic acid and *p*-coumaric acid. However, Nara et al. indicated that ferulic acid was the predominant bound phenolic acid in potato peel [35]. Albishi et al. reported the caffeic acid (0.1 mg/100 g DW) and *p*-coumaric acid (0.1 mg/100 g DW) were detected in bound phenolics in purple potato samples [13], but content reported in Albishi et al. was much lower than that in this study, which may be due to different extraction methods and possibly different genotypes.

### 3.4. Correlation Between Individual Phenolic Acid Content and TPC and Antioxidant Activity

To shed light on the possible contribution of the individual phenolic acid to the TPC and total antioxidant activity, the correlation coefficients among these parameters are listed in Table 5. Among free fraction of all potato accessions, chlorogenic, neochlorogenic, cryptochlorogenic, *p*-coumaric and ferulic acids were positively correlated with TPC and antioxidant activity, whereas caffeic acid was positively correlated with antioxidant activity but not correlated with TPC. Regarding the yellow and white flesh potatoes, chlorogenic acid content had positive correlation with TPC and antioxidant activity, caffeic acid content had positive correlation with DPPH and ABTS values, and *p*-coumaric acid content was positively correlated to the TPC. Regarding the colored potato accessions, chlorogenic acid and ferulic acid were positively correlated with the TPC and antioxidant activity in the free fractions, whereas neochlorogenic acid, cryptochlorogenic acid and *p*-coumaric acid were not correlated with TPC and antioxidant activity. Nara et al. indicated the chlorogenic acid and caffeic acid in the free fraction from the peel were highly correlated with the DPPH radical scavenging activity [35]. Reddivari et al. used standards to estimate the contribution of individual phenolic acid to antioxidant activity in potatoes and reported that chlorogenic acid contributed 28% to 45% to antioxidant activity, followed by gallic acid, catechin and caffeic acid [30].

Among bound fraction of colored potato accessions, caffeic acid and *p*-coumaric acid were significantly positively correlated with the TPC and antioxidant activity. The correlation of ferulic acid in bound fraction was different from that in free fraction of colored potato accessions. It was not positively correlated with the TPC and antioxidant activity of bound fraction.

### 3.5. TAC

Anthocyanins were only identified in the four colored potato samples and were quantified by HPLC-MS/MS and the pH differential method (Table 6). Pelargonidin-3-*p*-coumaorylrutinoside-5-glucoside was detected in red flesh potatoes PT11 and PT14 (Table 6). Petunidin-2-*p*-coumarylrutinoside-5-glucoside and pelargonidin-3-feruloylrutinoside-5-glucoside were detected in purple flesh potatoes (PT13 and PT18). Nemś et al. showed that pelargonidin derivatives were the most abundant anthocyanins in the red-fleshed potatoes and petunidin and malvidin were the main anthocyanins in the purple-fleshed potatoes [20].

The value of TAC in potato powder, expressed as cyanidin 3-glucoside equivalent, ranged from 1.13 mg/100 g (PT14) to 20.80 mg/100 g (PT13). Similar results of TAC in potato flour were reported by Nemś et al. [20]. According to the extraction method, anthocyanins were extracted in the free fraction. In our study, anthocyanin content was not significantly positively correlated with free TPC (*r* = 0.693, *p* > 0.05) and bound TPC (*r* = 0.325, *p* > 0.05) (Table 7), although PT13 had the highest anthocyanin content and the highest free TPC.

## 4. Conclusions

The TPC, phenolic acids content and antioxidant activities in the free and bound fractions of potatoes with different flesh color were systematically assayed. The total free TPC was significantly higher than the bound TPC in all cultivars. Colored potatoes had higher TPC than yellow and white flesh potatoes, which also had higher antioxidant capacity than yellow and white potatoes. The free and bound TPC were strongly positively correlated with antioxidant activity of free and bound fractions. Chlorogenic acid, neochlorogenic acid, cryptochlorogenic acid, caffeic acid, *p*-coumaric acid and ferulic acid were detected in the free fraction. Chlorogenic acid was the most abundant phenolic acid and accounted for 35.21–81.78% of the total free phenolic acid contents. The caffeic acid, *p*-coumaric acid and ferulic acid were detected in the bound fraction of the colored potatoes. In the free fraction, the content of each individual phenolic acid was positively correlated with antioxidant activity. In the bound fraction, caffeic acid and *p*-coumaric acid contents were positively correlated with antioxidant activity. The TAC of purple flesh potato was higher than red flesh potato. The TAC was not positively correlated with free TPC. This study promotes the further understanding of the correlations among the TPC, antioxidant activity and phenolic acid content in the free and bound fraction in potatoes with different flesh color and provides the theoretical basis for breeders to screen higher functional varieties based on the correlations reported in this study.

## Figures and Tables

**Table 1:** TPC, DPPH and ABTS radical scavenging activities of free and bound fractions of 14 potatoes.

Code	Accession	Flesh Color	TPC		DPPH		ABTS	
			Bound	Free	Bound	Free	Bound	Free
PT10	Weiyu 5	white	6.14 ± 0.11 E	13.98 ± 0.17 EFG	26.13 ± 1.75 D	58.56 ± 0.66 F	6.40 ± 1.15 EF	31.22 ± 2.20 FG
PT12	Helan 15	white	5.31 ± 0.19 EF	19.91 ± 0.41 D	20.05 ± 1.32 E	67.71 ± 7.99 EF	17.96 ± 0.95 CD	45.63 ± 3.40 EF
PT28	Dongnong 303	white	3.05 ± 0.50 FG	11.43 ± 0.88 GH	18.56 ± 0.52 E	62.86 ± 5.32 F	3.79 ± 0.03 F	36.25 ± 4.91 FG
PT05	Haerbin2	yellow	3.76 ± 0.45 FG	17.21 ± 0.51 DE	7.68 ± 0.87 FG	59.45 ± 1.09 F	0.64 ± 0.23 F	45.71 ± 1.68 EF
PT29	Kexin 13	yellow	3.41 ± 0.98 FG	8.77 ± 0.38 H	7.03 ± 0.66 G	21.76 ± 0.70 H	3.83 ± 0.87 F	21.23 ± 0.58 G
PT30	Helan	yellow	2.63 ± 0.09 G	12.93 ± 0.23 FG	7.35 ± 0.67 G	43.69 ± 0.24 G	4.60 ± 0.69 F	45.29 ± 1.08 EF
PT34	Zhongshu 3	yellow	3.53 ± 0.03F G	12.21 ± 1.49 GH	9.68 ± 0.40 FG	48.31 ± 2.12 G	2.67 ± 1.99 F	31.51 ± 4.79 FG
PT35	Zhongshu 5	yellow	4.06 ± 0.39 F	11.84 ± 2.97 GH	10.92 ± 0.72 F	42.23 ± 12.04 G	13.21 ± 0.65 DE	39.13 ± 5.64 EF
PT36	Zhongshu 6	yellow	5.36 ± 0.50 E	16.63 ± 1.16 DEF	26.74 ± 0.15 D	75.93 ± 4.58 E	24.72 ± 1.02 C	61.44 ± 4.30 D
PT38	Zhongshu 13	yellow	4.16 ± 0.02 F	16.52 ± 0.41 DEF	18.77 ± 2.27 E	68.01 ± 0.03E F	13.74 ± 1.46 DE	53.63 ± 1.68 DE
PT11	Hongyu	red	33.41 ± 0.02 B	51.36 ± 2.57 C	128.08 ± 4.23 B	206.65 ± 6.85 D	116.74 ± 2.18 A	183.01 ± 6.42 C
PT14	Lijiang	red	7.72 ± 0.70 D	65.18 ± 0.30 B	29.20 ± 1.02 D	240.18 ± 1.80 C	8.38 ± 1.06 EF	233.38 ± 5.43 B
PT13	Heijinggang	purple	19.33 ± 0.05 C	73.60 ± 4.99 A	62.67 ± 0.49 C	278.33 ± 0.49 A	53.32 ± 2.26 B	309.48 ± 8.53 A
PT18	Hemeiren	purple	40.45 ± 1.38 A	70.67 ± 0.86 A	132.75 ± 0.43 A	265.8 ± 0.92 B	124.49 ± 13.10 A	300.47 ± 21.76 A

The results (means ± SD) of TPC are presented as mg gallic acid equivalent/100 g potato powder, and the results (means ± SD) of DPPH and ABTS are presented as μmol Trolox equivalent/100 g potato powder. The values in each column with different capital letters (A–H) are significantly different (*p* < 0.05). TPC, total phenolic content. DPPH, 1,1-diphenyl-2-picrylhydrazyl radical 2,2-diphenyl-1-(2,4,6-trinitrophenyl) hydrazyl. ABTS, 2,2-azino-bis-3-ethylbenzothiazoline-6-sulphonic acid diammonium salt.

**Table 2:** Pearson pair-wise correlations between TPC, DPPH and ABTS in the free and bound fraction of the 14 potato cultivars.

Fraction	Traits	Free Fraction			Bound Fraction		
		TPC	DPPH	ABTS	TPC	DPPH	ABTS
Free fraction	TPC	1					
	DPPH	0.993 **	1				
	ABTS	0.989 **	0.989**	1			
Bound fraction	TPC	0.775 **	0.791 **	0.790 **	1		
	DPPH	0.753 **	0.779 **	0.759 **	0.989 **	1	
	ABTS	0.705 **	0.726 **	0.718 **	0.981 **	0.985 **	1

** indicate significance at *p* < 0.01.

**Table 3:** Individual phenolic acid content in the free fraction of different potato cultivars.

Cultivars	Flesh Color	nCGA	CGA	cCGA	CA	*p*-CA	FA
PT10	white	0.11 ± 0.01 F	4.37 ± 0.12 E	0.86 ± 0.03 D	1.86 ± 0.01 B	0.18 ± 0.00 A	0.92 ± 0.15 BC
PT12	white	0.37 ± 0.06 EF	6.83 ± 1.24 E	1.68 ± 0.33 CD	0.95 ± 0.00 B	0.60 ± 0.02 A	4.76 ± 2.56 ABC
PT28	white	0.11 ± 0.02 F	3.83 ± 0.66 E	0.67 ± 0.10 D	1.02 ± 0.17 B	0.31 ± 0.03 A	0.70 ± 0.01 BC
PT05	yellow	0.90 ± 0.04 DE	5.34 ± 0.47 E	1.77 ± 0.21 CD	2.23 ± 0.27 B	0.37 ± 0.02 A	4.93 ± 0.67 ABC
PT29	yellow	0.04 ± 0.01 F	2.06 ± 0.21 E	0.36 ± 0.04 D	nd	nd	3.05 ± 1.08 ABC
PT30	yellow	0.80 ± 0.17 DE	4.52 ± 0.26 E	0.35 ± 0.09 D	1.44 ± 0.29 B	0.14 ± 0.00 A	2.38 ± 0.17 ABC
PT34	yellow	0.56 ± 0.07 DEF	5.33 ± 0.80 E	2.85 ± 0.50 C	1.42 ± 0.14 B	nd	0.14 ± 0.07 C
PT35	yellow	0.19 ± 0.13 F	4.45 ± 0.97 E	0.95 ± 0.23 D	1.33 ± 0.21 B	0.37 ± 0.03 A	0.78 ± 0.34 BC
PT36	yellow	0.19 ± 0.03 F	6.85 ± 0.62 E	1.33 ± 0.14 CD	6.66 ± 1.48 A	nd	0.20 ± 0.01 C
PT38	yellow	0.39 ± 0.04 EF	7.94 ± 0.16 E	1.82 ± 0.05 CD	2.63 ± 0.24 B	nd	0.03 ± 0.04 C
PT11	red	1.58 ± 0.09 BC	42.24 ± 1.81 D	6.17 ± 0.43 B	nd	0.19 ± 0.00 A	1.66 ± 0.91 BC
PT14	red	1.79 ± 0.35 B	55.43 ± 0.7 C	9.74 ± 0.50 A	nd	0.50 ± 0.11 A	6.00 ± 2.54 ABC
PT13	purple	3.56 ± 0.36 A	79.91 ± 8.47 A	11.14 ± 1.03 A	nd	0.66 ± 0.26 A	9.06 ± 5.50 A
PT18	purple	1.04 ± 0.01 CD	69.15 ± 3.21 B	5.29 ± 0.13 B	nd	0.44 ± 0.03 A	8.04 ± 1.52 AB

The results (means ± SD) are presented as mg/100 g potato powder, and values in each column with different capital letters (A–F) are significantly different (*p* < 0.05). CGA, chlorogenic acid; nCGA, neochlorogenic acid; cCGA, cryptochlorogenic acid; CA, caffeic acid; *p*-CA, *p*-coumaric acid; FA, ferulic acid; nd, not detected.

**Table 4:** Individual phenolic acid content in the bound fraction of colored flesh potatoes.

Cultivars	CA	*p*-CA	FA
PT11	31.34 ± 1.43 A	10.11 ± 0.00 B	0.76 ± 1.13 C
PT13	13.46 ± 0.31 C	7.88 ± 0.20 C	5.57 ± 1.27 A
PT14	3.50 ± 0.22 D	1.07 ± 0.01 D	1.83 ± 0.41 BC
PT18	25.80 ± 2.95 B	16.19 ± 1.13 A	3.43 ± 0.25 BC

The results are presented as mg/100 g potato powder, and values in each column with different capital letters (A–D) are significantly different (*p* < 0.05). CA, caffeic acid; *p*-CA, *p*-coumaric acid; FA, ferulic acid; nd, not detected.

**Table 5:** Correlation coefficients between different phenolic acids and TPC and the antioxidant activity.

Phenolic Acids	All Potatoes (*n* = 14)			Red and Purple Fleshed Potatoes (*n* = 4)			Yellow and White Fleshed Potatoes (*n* = 10)		
	TPC	DPPH	ABTS	TPC	DPPH	ABTS	TPC	DPPH	ABTS
Free fraction									
nCGA	0.826 **	0.817 **	0.82 2**	0.428	0.466	0.382	0.327	0.094	0.309
CGA	0.989 **	0.984 **	0.995 **	0.940 **	0.955 **	0.961 **	0.768 **	0.809 **	0.796 **
cCGA	0.922 **	0.917 **	0.898 **	0.447	0.397	0.279	0.43	0.403	0.237
CA	0.451	0.654 **	0.778 **	nd	nd	nd	0.451	0.654 **	0.778 **
*p*-CA	0.526 *	0.486 *	0.488 *	0.626	0.484	0.443	0.774 **	0.529	0.386
FA	0.725 **	0.675 **	0.722 **	0.855 **	0.773 *	0.794 *	0.317	−0.098	−0.093
Bound fraction									
CA	nd	nd	nd	0.919 **	0.970 **	0.971 **	nd	nd	nd
*p*-CA	nd	nd	nd	0.959 **	0.904 **	0.926 **	nd	nd	nd
FA	nd	nd	nd	−0.068	−0.204	−0.129	nd	nd	nd

* and ** indicate significance at *p* ≤ 0.05 and *p* ≤ 0.01, respectively. CGA, chlorogenic acid; nCGA, neochlorogenic acid; cCGA, cryptochlorogenic acid; CA, caffeic acid; *p*-CA, *p*-coumaric acid; FA, ferulic acid; nd, not detected.

**Table 6:** Mass spectra (*m/z*) of detected anthocyanins and total anthocyanin content in colored potatoes.

Cultivars	Flesh Color	Retain Time	[M + H]^+^ (Frag. MS^2^ *m/z*)	Compound	TAC
PT11	red	13.07	887, 214 (271, 433, 725)	Pelargonidin-3-p-coumaorylrutinoside-5-glucoside	4.95 ± 0.12 C
PT14	red	13.03	887, 214, 279 (271, 433, 725)	Pelargonidin-3-*p*-coumaorylrutinoside-5-glucoside	1.13 ± 0.13 D
PT13	purple	12.27	933, 369, 417 (317,479,771)	Petunidin-2-*p*-coumarylrutinoside-5-glucoside	20.80 ± 1.12 A
		13.35	917, 214 (301, 463, 755)	Pelargonidin-3-feruloylrutinoside-5-glucoside	
PT18	purple	12.33	933, 369, 417 (317, 479, 771)	Petunidin-2-*p*-coumarylrutinoside-5-glucoside	14.12 ± 1.37 B
		13.40	917, 214 (301, 463, 755)	Pelargonidin-3-feruloylrutinoside-5-glucoside	

The results of TAC are presented as mg cyanidin-3-glucoside equivalent/100 g potato powder, and values in each column with different capital letters (A–D) are significantly different (*p* < 0.05). TAC, total anthocyanin content.

**Table 7:** Pearson pair-wise correlations between total anthocyanin content (TAC), total phenolic content (TPC) in the free and bound fraction in the four colored potatoes.

Traits	Free TPC	Bound TPC	TAC
Free TPC	1		
bound TPC	−0.179 ^a^	1	
TAC	0.693 ^a^	0.325 ^a^	1

^a^ significant at *p* > 0.05.

## References

[1] Ahmed S., Zhou X., Pang Y., Xu Y., Tong C., Bao J.S. (2018). Genetic diversity of potato genotypes estimated by starch physicochemical properties and microsatellite markers. Food Chem..

[2] Ezekiel R., Singh N., Sharma S., Kaur A. (2013). Beneficial phytochemicals in potato—A review. Food Res. Int..

[3] Singh P.P., Saldaña M.D. (2011). Subcritical water extraction of phenolic compounds from potato peel. Food Res. Int..

[4] FAOSTAT Food and Agriculture Organization Statistical Database. http://faostat.fao.org/.

[5] Camire M.E., Kubow S., Donnelly D.J. (2009). Potatoes and human health. Crit. Rev. Food Sci. Nutr..

[6] Burgos G., Auqui S., Amoros W., Salas E., Bonierbale M. (2009). Ascorbic acid concentration of native Andean potato varieties as affected by environment, cooking and storage. J. Food Compos. Anal..

[7] Calliope S.R., Lobo M.O., Sammán N.C. (2018). Biodiversity of Andean potatoes: Morphological, nutritional and functional characterization. Food Chem..

[8] Friedman M. (1997). Chemistry, biochemistry, and dietary role of potato polyphenols. A review. J. Agric. Food Chem..

[9] Burlingame B., Mouillé B., Charrondiere R. (2009). Nutrients, bioactive non-nutrients and anti-nutrients in potatoes. J. Food Compos. Anal..

[10] Ramamurthy M.S., Maiti B., Thomas P., Nair P.M. (1992). High-performance liquid chromatography determination of phenolic acids in potato tubers (*Solanum tuberosum*) during wound healing. J. Agric. Food Chem..

[11] Im H.W., Suh B.S., Lee S.U., Kozukue N., Ohnisi-Kameyama M., Levin C.E., Friedman M. (2008). Analysis of phenolic compounds by high-performance liquid chromatography and liquid chromatography/mass spectrometry in potato plant flowers, leaves, stems, and tubers and in home-processed potatoes. J. Agric. Food Chem..

[12] Xu X., Li W., Lu Z., Beta T., Hydamaka A.W. (2009). Phenolic content, composition, antioxidant activity, and their changes during domestic cooking of potatoes. J. Agric. Food Chem..

[13] Albishi T., John J.A., Al-Khalifa A.S., Shahidi F. (2013). Phenolic content and antioxidant activities of selected potato varieties and their processing by-products. J. Funct. Foods.

[14] Kim J., Soh S.Y., Bae H., Nam S.Y. (2019). Antioxidant and phenolic contents in potatoes (*Solanum tuberosum* L.) and micropropagated potatoes. Appl. Biol. Chem..

[15] Nardini M., Ghiselli A. (2004). Determination of free and bound phenolic acids in beer. Food Chem..

[16] Chu Y.F., Sun J.I.E., Wu X., Liu R.H. (2002). Antioxidant and antiproliferative activities of common vegetables. J. Agric. Food Chem..

[17] Eichhorn S., Winterhalter P. (2005). Anthocyanins from pigmented potato (*Solanum tuberosum* L.) varieties. Food Res. Int..

[18] Fossen T., Andersen Ø.M. (2000). Anthocyanins from tubers and shoots of the purple potato. Solanum tuberosum. J. Hortic. Sci. Biotechnol..

[19] Harborne J.B. (1960). Plant polyphenols. 1. Anthocyanin production in the cultivated potato. Biochem. J..

[20] Nemś A., Pęksa A., Kucharska A.Z., Sokół-Łętowska A., Kita A., Drożdż W., Hamouz K. (2015). Anthocyanin and antioxidant activity of snacks with coloured potato. Food Chem..

[21] Pang Y., Ahmed S., Xu Y., Beta T., Zhu Z., Shao Y., Bao J. (2018). Bound phenolic compounds and antioxidant properties of whole grain and bran of white, red and black rice. Food Chem..

[22] Shen Y., Jin L., Xiao P., Lu Y., Bao J. (2009). Total phenolics, flavonoids, antioxidant capacity in rice grain and their relations to grain color, size and weight. J. Cereal Sci..

[23] Yamaguchi T., Takamura H., Matoba T., Terao J. (1998). HPLC method for evaluation of the free radical-scavenging activity of foods by using 1, 1-diphenyl-2-picrylhydrazyl. Biosci. Biotechnol. Biochem..

[24] Re R., Pellegrini N., Proteggente A., Pannala A., Yang M., Rice-Evans C. (1999). Antioxidant activity applying an improved ABTS radical cation decolorization assay. Free Radic. Biol. Med..

[25] Fuleki T., Francis F.J. (1968). Quantitative methods for anthocyanins. J. Food Sci..

[26] Hosseinian F.S., Li W., Beta T. (2008). Measurement of anthocyanins and other phytochemicals in purple wheat. Food Chem..

[27] Shao Y., Xu F., Sun X., Bao J., Beta T. (2014). Phenolic acids, anthocyanins, and antioxidant capacity in rice (*Oryza sativa* L.) grains at four stages of development after flowering. Food Chem..

[28] Bonoli M., Verardo V., Marconi E., Caboni M.F. (2004). Antioxidant phenols in barley (*Hordeum vulgare* L.) flour: Comparative spectrophotometric study among extraction methods of free and bound phenolic compounds. J. Agric. Food Chem..

[29] Pillai S.S., Navarre D.A., Bamberg J. (2013). Analysis of polyphenols, anthocyanins and carotenoids in tubers from *Solanum tuberosum* group Phureja, Stenotomum and Andigena. Am. J. Potato Res..

[30] Reddivari L., Hale A.L., Miller J.C. (2007). Determination of phenolic content, composition and their contribution to antioxidant activity in specialty potato selections. Am. J. Potato Res..

[31] Reddivari L., Hale A.L., Miller J.C. (2007). Genotype, location, and year influence antioxidant activity, carotenoid content, phenolic content, and composition in specialty potatoes. J. Agric. Food Chem..

[32] Hesam F., Balali G.R., Tehrani R.T. (2012). Evaluation of antioxidant activity of three common potato (*Solanum tuberosum*) cultivars in Iran. Avicenna J. Phytomed..

[33] Rojas-Padilla C.R., Vasquez-Villalobos V.J., Vital C.E., Rojas J.C., Rios N.H., Lujan A.P., Ninaquispe V.P., Espinoza M.S. (2019). Phenolic compounds in native potato (*Solanum tuberosum* L.) cooking water, with potential antioxidant activity. Food Sci. Technol..

[34] Shao Y., Bao J. (2015). Polyphenols in whole rice grain: Genetic diversity and health benefits. Food Chem..

[35] Nara K., Miyoshi T., Honma T., Koga H. (2006). Antioxidative activity of bound-form phenolics in potato peel. Biosci. Biotechnol. Biochem..

